# C1q tumor necrosis factor α-related protein isoform 5 attenuates palmitate-induced DNA fragmentation in myocytes through an AMPK-dependent mechanism

**DOI:** 10.1016/j.dib.2015.10.035

**Published:** 2015-11-06

**Authors:** Won-Mo Yang, Kyung-Ho Min, Wan Lee

**Affiliations:** a Department of Biochemistry, Dongguk University College of Medicine, Gyeongju 780-714, Republic of Korea; b Endocrine Channelopathy, Channelopathy Research Center, Dongguk University College of Medicine, Goyang 410-773, Republic of Korea

**Keywords:** CTRP5, Palmitate, DNA fragmentation, Caspase-3, MTT assay, Myocytes

## Abstract

This article reports the data for the effects of C1q tumor necrosis factor α-related protein isoform 5 (CTRP5) on the palmitate-induced apoptosis in myocytes. The data obtained from in vitro cultured myocytes shows that the cellular treatment with the globular domain of CTRP5 (gCTRP5) significantly inhibits the palmitate-induced MTT reduction, caspase-3 activation, and DNA fragmentation in a time-dependent manner. The data presented in this article also shows that AraA, an inhibitor of AMPK, almost completely abolished the protective effect of gCTRP5 on the DNA fragmentation induced by palmitate in myocytes. Interpretation of our data and further extensive insights into the protective role of CTRP5 in palmitate-induced apoptosis in myocytes can be found in Yang and Lee (2014) [1].

**Specifications Table**

**Table:** 

Subject area	*Cell biology, biochemistry*
More specific subject area	*Obesity, apoptosis, metabolism, cytokine*
Type of data	*Figure and text*
How data was acquired	*Analysis of MTT reduction, caspase-3 activation and DNA fragmentation*
Data format	*Analyzed*
Experimental factors	*L6 GLUT4myc myocytes were treated with palmitate, gCTRP5 or AraA.*
Experimental features	*L6 GLUT4myc myocytes were incubated with palmitate (0.5 mM) in the presence or absence of gCTRP5 (5 μg/ml) for 0–18 h. For inhibition of AMPK, 2 mM of AraA was co-treated during the incubation.*
Data source location	*Dongguk University School of Medicine, Gyeongju 780–714, Korea*
Data accessibility	*The data are supplied with this article*

**Value of the data**

• The protective effect of CTRP5, an adipokine paralog, on palmitate-induced cytotoxicity, activation of caspase-3, and DNA fragmentation is unveiled in myocytes.
• The functional analysis of CTRP5 provides an insight into the regulation of cell survival and apoptosis by the fatty acids accumulation and C1q complement-related cytokines.
• This data allows to predict biological significance of other cytokines containing C1q complement domain in various diseases associated with obesity and dyslipidemia*.*

## 1 Data

C1q tumor necrosis factor α-related protein isoform 5 (CTRP5), a member of the CTRP family, has recently been identified as a highly conserved family of adiponectin paralog [2]. Adiponectin is an abundant adipokine involved in the regulation of energy metabolism, such as fatty acid oxidation and glucose utilization [3]. Similar to adiponectin, we have previously shown that the globular domain of CTRP5 (gCTRP5) activates AMPK, which subsequently stimulates fatty acid oxidation and glucose uptake in myocytes [4].

**Fig. 1:**
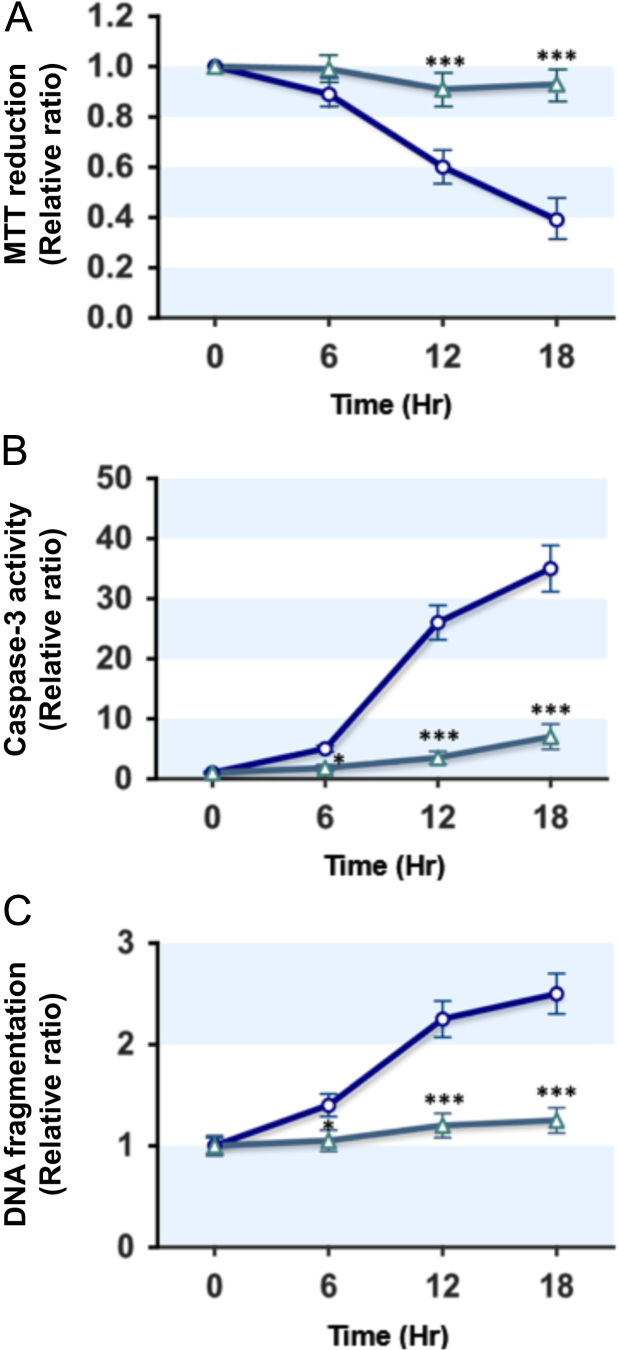
*gCTRP5 prevents myocytes from palmitate-induced cytotoxicity, caspase-3 activation and DNA fragmentation.* L6 GLUT4myc myocytes were incubated with palmitate (0.5 mM) in the presence (triangle) or absence (circle) of gCTRP5 (5 μg/ml) for 0–18 h. The values are expressed as the relative ratio, where the level of zero time control was set to one. Values are expressed as means±SEM of three independent experiments; *, *P*<0.05; ***, *P*<0.001.

It has been reported that a high concentration of palmitate, the most abundant dietary saturated fatty acid (SFA), induces apoptosis and insulin resistance in skeletal muscle cells through the activation of PKC and NFκ-B, increase of oxidative stress and mitochondrial dysfunction, *etc*. [5], [6], [7], [8]. Therefore, we analyzed the protective effect of CTRP5 on SFA-induced lipotoxicity, such as cell viability and apoptosis, in myocytes. We treated L6 GLUT4myc myocytes with 0.5 mM of palmitate for 0–18 h and the effects of palmitate on cell viability and apoptosis were examined. As shown in Fig. 1, we found that the cellular treatment with palmitate in L6 GLUT4myc myocytes decreased cell viability, as assessed by MTT reduction, and increased caspase-3 activity and DNA fragmentation, in a time-dependent manner. Interestingly, the treatment of gCTRP5 (5 ug/ml) significantly rescued the palmitate-induced cytotoxicity, caspase-3 activation and DNA fragmentation in L6 GLUT4myc myocytes (Fig. 1 A–C).

**Fig. 2:**
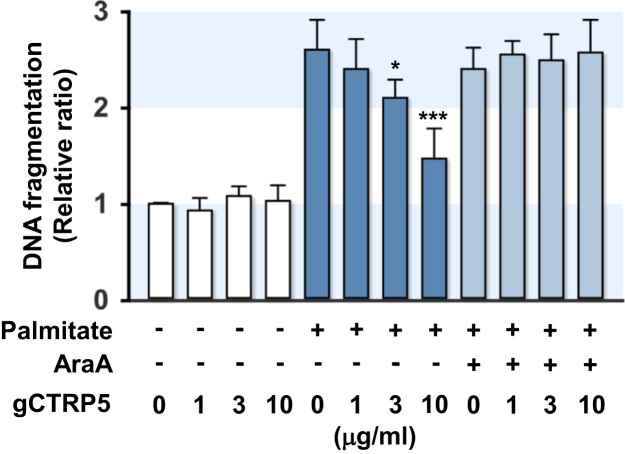
*Inhibition of AMPK abolished the effect of gCTRP5 against palmitate-induced DNA fragmentation in myocytes.* L6 GLUT4myc myocytes were incubated in the presence or absence of palmitate (0.5 mM for 18 h) and/or globular domain of human CTRP5 (gCTRP5, 5 µg/ml). For inhibition of AMPK, 2 mM of AraA was co-treated during the incubation. DNA fragmentation was measured by an ELISA assay. The values are expressed as the relative ratio, where the level of the untreated control was set to one. Values are expressed as means±SEM of three independent experiments; *, *P*<0.05; ***, *P*<0.001.

We next examined whether protective effect of gCTRP5 in palmitate-induced DNA fragmentation is dependent on AMPK activation in myocytes, because we have previously shown that the anti-apoptotic effect of gCTRP5 is dependent on AMPK [1]. As we expected, gCTRP5 significantly inhibited the palmitate-induced DNA fragmentation in a dose-dependent manner, whereas gCTRP5 did not affect DNA fragmentation in the control cells (Fig. 2). Moreover, it is noteworthy that AraA, an inhibitor of AMPK, almost completely abolished the protective effect of gCTRP5 on the DNA fragmentation induced by palmitate in myocytes (Fig. 2), indicating that the anti-apoptotic effect of gCTRP5 in palmitate-treated cells is dependent on AMPK activation.

## 2 **Experimental design, materials and methods**

### 2.1 Cell culture and palmitate treatment

L6 GLUT4myc cells, immortalized rat skeletal muscle cells stably expressing GLUT4 containing an exofacial myc epitope [9], were generously provided by Dr. Amira Klip (the Hospital for Sick Children, Toronto, Ontario, Canada). The cells differentiate normally from myoblasts to myotubes, when they were harvested in MEM alpha supplemented with 10% FBS and 1% penicillin–streptomycin [10]. For the palmitate treatment, the cells were incubated for 6–18 h with BSA or BSA conjugated-palmitate (0.5 mM) in culture media. If necessary, recombinant gCTRP5 (5 μg/ml) and/or adenine 9-D-arabinofuranoside (AraA, 2 mM), an AMPK inhibitor, were treated with or without palmitate (0.5 mM).

### 2.2 Analysis of cytotoxicity

Palmitate-induced cytotoxicity in L6 GLUT4myc myocytes was measured by MTT (3-[4,5-dimethylthiazol-2-yl]-2,5 diphenyl tetrazolium bromide) assay. Briefly, the cells were incubation in media containing 1.2 mM MTT solution for 4 h. Following incubation, 40% DMSO was added to each well, and the absorbance was measured at 540 nm. The reduction of MTT was used to determine viability of cells.

### 2.3 Determination of cell apoptosis

Apoptosis was assessed by measurement of cellular caspase-3 activity, which is based on the hydrolysis of the peptide substrate of caspase-3, using colorimetric methods according to the manufacturer׳s instructions (R&D Systems).

### 2.4 Analysis of DNA fragmentation

DNA fragments were quantified by the Cell Death Detection ELISA Plus (Roche Molecular Biochemicals, Indianapolis, Indiana, USA). In brief, myocytes were rinsed with PBS three times in order to remove nucleosomes leaked out from necrotic cells and then incubated with the lysis buffer for 30 min. The supernatant containing mono- and oligo-nucleosomes from the cytoplasmic fraction of apoptotic cells was used for further analyses.

### 2.5 Statistical analysis

Values are expressed as the mean±SEM from at least four independent experiments. Where applicable, the significance of the differences was determined using a Student׳s *t* test for the unpaired data.
